# Does Environmental Regulation Affect Export Quality? Theory and Evidence from China

**DOI:** 10.3390/ijerph17218237

**Published:** 2020-11-07

**Authors:** Jing Xie, Qi Sun, Shaohong Wang, Xiaoping Li, Fei Fan

**Affiliations:** 1 School of Economics, Zhongnan University of Economics and Law, Wuhan 430073, China; xj@zuel.edu.cn (J.X.); qisun@stu.zuel.edu.cn (Q.S.); shw0123@stu.zuel.edu.cn (S.W.); chineselixp@zuel.edu.cn (X.L.); 2 Institute of Central China Development, Wuhan University, Wuhan 430072, China; 3 Institute of Regional and Urban-Rural Development, Wuhan University, Wuhan 430072, China

**Keywords:** environmental regulation, export quality, quality upgrading, process productivity, product productivity

## Abstract

Most studies focus on the empirical investigation of the relationship between environment and trade, but they lack a systematic theoretical framework. To fill this gap, this study constructs an analytical framework of export competitiveness from the perspective of product quality, and reveals the theoretical mechanism of environmental regulation affecting export quality. We empirically examine the impact of environmental regulation on the export quality of China’s manufacturing industry, as well as its possible mechanism. Our findings show that environmental regulation can significantly promote the export quality upgrading of the manufacturing industry and that process and product productivity are two possible channels through which such regulation affects export quality, although their mediating effects are in opposite directions. The mediating effect of product productivity is greater than that of process productivity, indicating that environmental regulation mainly has an innovation offset effect on China’s manufacturing industry. For pollution-intensive industries, environmental regulation plays a significant promoting role through the channel of product productivity, but, for clean industries, environmental regulation has an inhibitory effect through the channel of process productivity. These findings provide important enlightenment for the coordinated development of China’s ecological civilization and trade power.

## 1. Introduction

Since its accession to the World Trade Organization (WTO), China’s export trade has expanded rapidly, making it the world’s largest exporter in 2009. However, even though “Made in China” has achieved considerable market share in international competition, it has not yet been “winning by quality.” In other words, there is still a significant gap between China’s product quality and that of developed countries [1]. Consequently, low-quality and low-price exports cause persistent deterioration in China’s terms of trade. At present, with international competition undergoing a profound change, countries ought to seize the opportunity to rapidly improve export competitiveness, thereby moving closer to the center of the world stage and increasing the possibility of leading international trade regulation. The transformation and upgrading of China’s export trade need to improve product quality through technological innovation, thus cultivating new advantages in export competition.

However, along with the rapid development of its economy and trade, China has paid a heavy price for environmental pollution. In recent years, due to the frequent occurrences of an air-quality index over 500 (the maximum pollution level) in many regions, the social desirability of a beautiful ecological environment is becoming more and more urgent. Therefore, the Chinese government is attaching increasing importance to environmental protection. From “The 11th Five-Year Plan” to “The 13th Five-Year Plan,” the government has continuously strengthened the intensity of environmental regulation and implemented a high standard of environmental protection measures to improve the quality of the ecological environment. However, environmental regulation is a “double-edged sword” [2]. Will strengthening environmental governance inhibit industrial technological innovation? Can the enforcement of environmental regulation achieve a “win-win” situation between environmental quality improvement and export quality upgrading? The above issues are related to the coordinated development of China’s ecological civilization and trade power and are worthy of in-depth exploration.

There are numerous studies on the relationship between environmental regulation and export competitiveness, and two totally different views have emerged. The conventional view holds that the compliance cost of strict environmental regulation may squeeze out some budgets for technological innovation and, thus, weaken the long-term competitiveness of a country’s exports [3,4,5]. Specifically, in order to achieve cleaner production and meet environmental standards, enterprises have to spend more on controlling and reducing pollution [6,7,8]. The additional costs for environmental protection activities impose a new constraint on firms’ production decisions and operations management, thereby having an inhibiting effect on enterprise competitiveness [9,10,11]. However, other empirical studies show that there is no significant evidence that supports the conventional view [12,13,14]. To modify the conventional view, Porter [15] and Porter and Linde [16] proposed that the relationship between environmental regulation and firm competitiveness is complementary, rather than mutually exclusive, through the innovation offset effect, which is referred to as the “Porter hypothesis”. The implementation of strict environmental regulation means the emergence of new profit opportunities, which can stimulate domestic firms to conduct environmentally related innovation and technological design [17,18,19,20,21]. As a result, this may promote firm productivity and the international competitiveness of domestic industry [22,23,24].

In contrast, there are different findings from the empirical research on the validity of the Porter hypothesis. Gray and Shadbegian [25] used paper-making enterprise data from the United States, Lanoie et al. [26] utilized manufacturing industry data from Canada, and Greenstone et al. [27] employed manufacturing enterprise data from the United States to conclude that environmental regulation has led to the decline of domestic productivity and international competitiveness to some extent. Others have reached conclusions that are more nuanced. Cole and Elliott [28], Marconi [29], Nesta et al. [30], and Liu and Xie [2] argued that the impacts of environmental regulation on export competitiveness might be heterogeneous across different countries or industries. In addition, Rexhäuser and Rammer [31], Banerjee and Gupta [32], and Yuan and Zhang [33] found that different types of environmental regulation policies possibly had distinct effects on competitiveness.

One possible explanation for the differences is that the measure of export competitiveness in most literature is neither appropriate nor realistic. Researchers largely use the comparative advantage indexes based on traditional trade theory as proxies for export competitiveness, such as revealed comparative advantage, market share, net export, and trade competitiveness. However, these indexes essentially reflect the competitive advantage of a country or industry in terms of export volume. In recent decades, with the gradual deepening of economic globalization and vertical specialization, export volume no longer directly represents the comprehensive strength of a country’s or industry’s participation in international trade and specialization, as it is disproportionate to trade gains [2]. Consequently, the comparative advantage indexes largely used in the existing literature cannot realistically reflect a country’s export competitiveness.

In view of this, it is particularly necessary to explore a more accurate measure of export competitiveness. Currently, the “new-new” trade theory, represented by the models of heterogeneous firms, has been widely popularized and developed. This theory emphasizes that firms have heterogeneity not only in productivity [34] but also in product quality [35]. Product quality plays a key role as a determinant of one country’s export performance and trade gains, especially after allowing for the vertical product differentiation. In addition, a substantial amount of empirical evidence indicates that product quality contributes significantly to re-understanding the source of export competitiveness [1,36,37]. Compared with traditional comparative advantage, product quality upgrading is more important and indispensable to export success and sustainable development in the context of economic globalization and vertical specialization. Therefore, product quality may provide a new perspective from which to investigate the systematic relationship between environmental regulation and export competitiveness. Moreover, the existing literature has mostly focused on whether environmental regulation improves or weakens export competitiveness, providing some useful insights into Porter hypothesis, but it lacks a systematic theoretical framework. Relatively few studies have pointed to how environmental regulation affects export competitiveness, as well as which possible channels are effective. Thus, further research is needed to fill the gap.

To supplement existing theoretical and empirical studies, this study constructs an analytical framework of export competitiveness from the perspective of product quality, intending to reveal the theoretical mechanism of environmental regulation affecting export quality. Further, we empirically examine the impact of environmental regulation on export quality for China’s manufacturing industry, as well as its possible mechanism. The findings show that, firstly, environmental regulation can significantly promote the export quality upgrading of the manufacturing industry, and China’s environmental regulation policies, to some extent, support the strong Porter hypothesis. Secondly, process and product productivity are two possible channels through which environmental regulation affects export quality, and their mediating effects have opposite directions. The mediating effect of product productivity is obviously greater than that of process productivity, indicating that environmental regulation mainly has an innovation offset effect on China’s manufacturing industry. Thirdly, the impact of environmental regulation on export quality and its mechanism show the significant heterogeneity of regulated industries. For pollution-intensive industries, environmental regulation plays a significant promoting role through the channel of product productivity. However, for clean industries, environmental regulation has a certain degree of inhibitory effect through the channel of process productivity.

The main contributions of this paper are reflected in the following two aspects. First, in a departure from the comparative advantage perspective largely used in previous literature, this study analyzes export competitiveness from the perspective of product quality, which can supplement existing research on the endogenous determinants of export competitiveness. Second, our study extends the stream of literature on the Porter hypothesis. We build a theoretical framework that reveals the internal relationship between environmental regulation and export quality and simultaneously provide empirical support for the Porter hypothesis. Third, our work contributes to the measure of product quality. Compared with the methods of Hallak and Schott [1] and Liao and Xie [38], we use the Elteto–Koves–Szulc (EKS) method to adjust the relevant indexes, which can not only simplify the calculation process but also increase the reliability and robustness of results.

This paper proceeds as follows. Section 2 presents the materials and methods used in our study, including our theoretical framework. Section 3 reports our empirical results. Section 4 discusses the implications of the results and identifies key implications and limitations of the research, and Section 5 concludes the paper.

## 2. Materials and Methods

### 2.1. Theoretical Framework

In this part, we mainly investigate the endogenous determinants of export competitiveness from the perspective of product quality based on the seminal study of Hallak and Sivadasan [37] on two dimensions of firm heterogeneity. Then, we analyze the mechanism of environmental regulation affecting the quality of export products.

#### 2.1.1. Endogenous Quality Choice of Firms

Considering the vertical differentiation of products, the utility function with constant elasticity of substitution is given by:

(1) $$U={\left\{\int_{j\in \Omega }{\left(\lambda_{j}q_{j}\right)}^{\frac{\sigma -1}{\sigma }}dj\right\}}^{\frac{\sigma }{\sigma -1}},$$

where $\lambda_{j}$ represents the quality of product *j*, $q_{j}$ denotes the consumption of product *j*, Ω is the consumer’s possible set of purchases, and σ>1 defines the elasticity of substitution between products. 

Following the majority of the international trade models, suppose *E* is the total expenditure in a country, and the country-level aggregate price index is represented by $P=\int_{j\in \Omega }p_{j}^{1-\sigma }\lambda_{j}^{\sigma -1}dj$. Therefore, *E*/*P* represents the overall market potential, which is exogenous from the perspective of individual enterprises. Then, the demand for product *j* of firm *i* is determined by:

(2) $$q_{ij}=\frac{\lambda_{ij}^{\sigma -1}}{p_{ij}^{\sigma }}\frac{E}{P},$$

where $p_{ij}$ represents the price of product *j* produced by firm *i*.

According to Hallak and Sivadasan [37], firm heterogeneity is extended from a single attribute into two dimensions, process productivity (ϕ) and product productivity (ξ), which can affect the marginal cost and fixed cost of a firm, respectively. Process productivity mainly reflects the difference between enterprises in the utilization efficiency of variable inputs, while product productivity emphatically focuses on the different ability of firms to improve product quality with given fixed inputs. Thus, marginal cost (*c*) and fixed cost (*F*) are set as follows:

(3) $$c_{ij}=\frac{\kappa }{\phi_{ij}}\lambda_{ij}^{\beta },$$

(4) $$F_{ij}=F_{0}+\frac{f}{\xi_{ij}}\lambda_{ij}^{\alpha },$$

where, $F_{0}$, and f are positive constants; β and α represent the quality elasticity of marginal cost and fixed cost, respectively; and 1>β>0 and α>0.

Combining the demand equation with the cost function above, we obtain the following profit function (the derivation is shown in Appendix A):

(5) $$\pi_{ij}=\frac{1}{\sigma }{\left(\frac{\lambda_{ij}}{p_{ij}}\right)}^{\sigma -1}\frac{E}{P}-F_{ij}-f_{x},$$

where $f_{x}$ represents the fixed trade costs. By maximizing the above profit function (Equation (5)), the solution for optimal product quality is obtained as follows:

(6) $$\lambda ={\left[\frac{1-\beta }{\alpha }{\left(\frac{\sigma -1}{\sigma }\right)}^{\sigma }{\left(\frac{\phi }{\kappa }\right)}^{\sigma -1}\frac{\xi }{f}\frac{E}{P}\right]}^{\frac{1}{\alpha^{\prime }}},$$

where $\alpha^{\prime }=\alpha -\left(1-\beta \right)\left(\sigma -1\right)>0$. Equation (6) implies that the optimal product quality of firms is endogenously determined by process productivity (ϕ) and product productivity (ξ). Furthermore, differentiating Equation (6) partially with respect to ϕ and *ξ*, we obtain $\frac{d\lambda }{d\phi }>0$ and $\frac{d\lambda }{d\xi }>0$. The two inequalities illustrate that the product quality of an enterprise increases with the improvement of process and product productivity.

#### 2.1.2. The Mechanism of Environmental Regulation Affecting Product Quality

Based on the analysis of the endogenous determinants of export quality, we further explore the mechanism of environmental regulation affecting export quality through the two channels of process and product productivity.

Some research shows that the compliance cost of environmental regulation may increase the factor inputs at a given output, reducing process productivity [25,27]. Therefore, the relationship between environmental regulation and process productivity can be expressed as $\frac{d\phi }{dER}<0$, where *ER* denotes the intensity of environmental regulation. Furthermore, according to the analysis of the preceding context, there is a positive correlation between process productivity and the quality of export products, so $\frac{d\lambda }{d\phi }>0$. Combining the two inequalities above, $\frac{d\lambda }{dER}=\frac{d\lambda }{d\phi }\cdot \frac{d\phi }{dER}<0$. In this case, it is demonstrated that environmental regulation can inhibit export quality upgrading through the process productivity channel.

Product productivity is defined as a firm’s effectiveness in improving product quality with given fixed expenditures [37]. In fact, the improvement of product quality cannot be divorced from the R&D investment and innovation activity. Many studies have found that the innovation offset effect of environmental regulation can stimulate firms to increase R&D investment and strengthen independent innovation capability [16,19], thus facilitating the improvement of product productivity [21,39]. Hence, the link between environmental regulation and product productivity can be expressed as $\frac{d\xi }{dER}>0$. Moreover, Equation (6) indicates that a firm’s product productivity is positively associated with the quality of its exports; therefore, $\frac{d\lambda }{d\xi }>0$. Similarly, it is not difficult to show that $\frac{d\lambda }{dER}=\frac{d\lambda }{d\xi }\cdot \frac{d\xi }{dER}>0$, which implies that environmental regulation can promote export quality upgrading through the product productivity channel.

In addition, to identify process and product productivity as two possible channels through which environmental regulation affects export quality, we can speculate that if the compliance cost of environmental regulation is more dominant, it may inhibit export quality upgrading to a certain extent. On the contrary, if the innovation offset effect of environmental regulation is more pronounced, it is conducive to quality upgrading.

### 2.2. Measuring Export Quality

Suppose there are *K* countries, each of which has *S* industries, and industry *s* has $Z_{s}$ products. According to Hallak and Schott [1], the impure price index (IPI, marked as $P_{st}^{ko}$) between country *k* and the benchmark country *o* in industry *s* is decomposed into the pure price index (PPI, marked as ${\tilde{P}}_{st}^{ko}$) and the export quality index ($\lambda_{st}^{ko}$), given by $P_{st}^{ko}={\tilde{P}}_{st}^{ko}\lambda_{st}^{ko}$. Furthermore, in view of the relationship between the net trade of country *k* in industry *s* and its PPI, which is given by ${\tilde{T}}_{st}^{k}=\beta_{0}+\gamma_{s}{\tilde{\ln P}}_{st}^{ko}+u_{st}$, the estimating equation of the export quality index can be obtained as follows:

(7) $${\tilde{T}}_{st}^{k}=\beta_{0}+\gamma_{s}{\hat{\ln P}}_{st}^{ko}-\gamma_{s}\ln\lambda_{st}^{ko}+u_{st},$$

where ${\tilde{T}}_{st}^{k}=(T_{st}^{k}-b_{s}T_{t}^{k})/E_{t}^{k}-b_{s}\tau_{st}^{k}$ is a polynomial of the net trade of country *k*. $T_{s}^{k}$ denotes the net trade of country *k* in industry *s*. $T^{k}$ represents the total net trade of country *k*. $E^{k}$ refers to the net expenditure of country *k* and equals the GDP minus the net trade. $\tau_{s}^{k}$ indicates the trade cost, measured by the improved gravity model of Novy [40]. $b_{s}$ is the proportion of the net expenditure of industry *s* in the total net expenditure of country *k*, and the net expenditure is the industry’s added value minus net trade. ${\hat{P}}_{st}^{ko}$ is the estimated value of the IPI. $\lambda_{st}^{ko}$ is the export quality index of country *k* relative to the benchmark country *o*. $u_{st}$ is the error term.

In order to address the endogeneity caused by the correlation between the regressor ($\lambda_{st}^{ko}$) and error term ($u_{st}$) in Equation (7), which includes the estimation error of IPI, we use the method of Hallak and Schott [1] to specify the linear time trend for product quality as:

(8) $$\ln\lambda_{st}^{ko}=\alpha_{0s}^{ko}+\alpha_{1s}^{ko}t+\varepsilon_{st}^{ko},$$

where $\alpha_{0s}^{ko}$ and $\alpha_{1s}^{ko}$ reflect a country fixed effect and time trend of export quality, respectively, and $\varepsilon_{st}^{ko}$ is the error of export quality deviating from time trend. By substituting Equation (8) into Equation (7), the final estimating equation of the export quality index is obtained as follows:

(9) $${\tilde{T}}_{st}^{k}=\beta_{0}+\gamma_{s}\ln{\hat{P}}_{st}^{ko}-\zeta_{0s}^{ko}-\zeta_{1s}^{ko}t+\upsilon_{st}^{ko},$$

where $\varsigma_{0s}^{ko}=\gamma_{s}\alpha_{0s}^{ko}$ and $\varsigma_{1s}^{ko}=\gamma_{s}\alpha_{1s}^{ko}$. $\upsilon_{st}=u_{st}-\gamma_{s}\varepsilon_{st}$ denotes the random error term.

Since the error term ($\upsilon_{st}$) in Equation (9) includes the deviation ($\varepsilon_{st}$) of export quality in Equation (8), there may still be a correlation between the error term ($\upsilon_{st}$) and the regressor (${\hat{P}}_{st}^{ko}$). In view of this, we take the real effective exchange rate of each country as an instrument for ${\hat{P}}_{st}^{ko}$ to control the endogeneity in Equation (9). Then, Equation (9) is estimated by two-stage least squares (2SLS). The estimated export quality index of country *k* relative to the benchmark country *o* in industry *s* is given by:

(10) $$\ln{\hat{\lambda }}_{st}^{ko}=-\frac{{\hat{\zeta }}_{0s}^{ko}+{\hat{\zeta }}_{1s}^{ko}t}{{\hat{\gamma }}_{s}},$$

In the steps above, the key link is the estimation of IPI, because it is unobservable. If the IPI can be estimated accurately, the export quality index is easy to calculate through Equations (9) and (10).

Hallak and Schott [1] employed the revealed preference to demonstrate that the IPI ($P_{s}^{kk^{\prime }}$) between country *k* and $k^{\prime }$ was bounded by the Paasche price index ($H_{s}^{kk^{\prime }}$) and the Laspeyres price index ($L_{s}^{kk^{\prime }}$). That is, $\ln H_{s}^{kk^{\prime }}\leq \ln P_{s}^{kk^{\prime }}\leq \ln L_{s}^{kk^{\prime }}$. Accordingly, they utilized the maximum likelihood method to estimate the IPI. However, this method has some limitations in the estimation of IPI. First, the assumptions of the method are too strict. The establishment of the likelihood function strictly requires that the error terms of the Laspeyres and Paasche price indexes not only coincide with the assumption of homoscedasticity but also be uncorrelated among the different country pairs. Second, the method is not quite flexible and concise enough for practical applications. Relaxing the above assumptions may cause the likelihood function to be difficult to deduce. Even though the strict assumptions of the error terms are all valid, the estimation process is already sufficiently complicated.

Liao and Xie [38] directly employed the Fisher index as the estimated value of the bilateral IPI, based on the excellent property that the Fisher index can reconcile the contradiction between the Laspeyres and Paasche price indexes. Furthermore, since the IPI is theoretically required to be transitive, there must be ${\hat{P}}_{s}^{kk^{\prime }}={\hat{P}}_{s}^{kx}/{\hat{P}}_{s}^{k^{\prime }x}$ for any given country *x*. However, the Fisher index is not transitive, so it is not suitable for the estimation of multilateral IPI. In view of this, we use the EKS method to adjust the Fisher index to meet the requirement of transitivity, thereby estimating the multilateral IPI.

First, we use the Fisher index to estimate the bilateral IPI between countries:

(11) Pb^skk′=∑zpzkqzk∑zpzk′qzk×∑zpzkqzk′∑zpzk′qzk′,

where *z* is the representative product of industry *s*, and $p_{z}^{k}$ and $q_{z}^{k}$ ($p_{z}^{k^{\prime }}$ and $q_{z}^{k^{\prime }}$) represent the export price and quantity of product *z* in country *k* (country $\;k^{’}$), respectively.

Second, we use the EKS method to adjust the bilateral IPI above, and the adjustment formula is as follows:

(12) P^skk′=[∏iCPb^skiPb^sk′i]1C,

where *C* is the number of common trade partners of country *k* and $k^{\prime }$. Using Equation (12), we easily obtain the estimated IPI, which satisfies the requirement of transitivity and can be compared multilaterally. Then, the estimate of the export quality index can be obtained from the estimation of Equations (9) and (10).

### 2.3. Data

The estimation above mainly involves three types of data. The first is trade data. The product-level export data are derived from the United Nations COMTRADA, in which each original record is at the 6-digit HS2002 level. The industry-level trade data are drawn from the OECD (Organization for Economic Co-operation and Development) Bilateral Trade Database by Industry and End-use (BTDIxE) at the 2-digit ISIC (International Standard Industrial Classification) Rev.4 level. The second is the data of GDP, value added of industry, and industrial output. The GDP of each country comes from the World Bank’s WDI database. The value added of industry and industrial output for each country are all drawn from the OECD Input-Output Table. The third is exchange rate data. The real effective exchange rates of countries that are used as instruments for the IPIs are computed by the nominal exchange rates and CPI (Consumer Price Index) series reported by the Penn World Table. In addition, considering that the United States is the largest importer in the world, this study selects the United States as the common destination country of other countries’ exports, which is conducive to obtaining sufficient product-level trade data for the calculation of the Fisher index. Moreover, in view of the data availability and the validity of the measurement, the data sets of this section are constructed from 1998 to 2015.

Due to the differences of statistical classification between the databases mentioned above, we need to adjust and integrate the different types of data. The detailed description of these steps is as follows. First, we use the detailed correspondence Table between HS2002 and ISIC Rev.3 provided by World Integrated Trade Solution (WITS) to aggregate the HS2002 6-digit codes to the ISIC Rev.3 4-digit and 2-digit codes, respectively. Then, using the correspondence Table between ISIC Rev.3 and Rev.4 provided by the OECD BTDIxE database, we match the ISIC Rev.3 4-digit and 2-digit codes with the corresponding ISIC Rev.4 codes. Accordingly, the product is defined at the ISIC Rev.4 4-digit level, and the sub-industry refers to the ISIC Rev.4 2-digit level. Second, the trade data of the 23 manufacturing sub-industries (excluding the repair and installation of machinery and equipment due to the absence of data) at the ISIC Rev.4 2-digit level are aggregated into 14 manufacturing industries as shown in Table 1, to be coincident with the industry-level value-added data. Third, for the sake of consistency, the initial industry-level value-added data obtained from the OECD Input-Output Table for 1998 to 2004 are adjusted from ISIC Rev.3 to ISIC Rev.4 due to a change in classification in the OECD Input-Output Table in 2005. In addition, in preparation for empirical analysis at a later stage, a close correspondence between ISIC Rev.4 and China’s National Industrial Classification (CNIC) 2002 at the 2-digit level is established in Table 1.

To ensure the accuracy of the estimation of IPIs, it is necessary to cleanse the initial data and remove the abnormal records. First, we delete the records that are incomplete at the product level. Second, we exclude the data in which the period of exports is less than three years at the product-destination country level. Third, we remove the records for which the unit value export price is either less than 1/7 or greater than 7 times the geometric average of that of all the export products in the same year at the product-destination country-year level. Fourth, we remove countries whose common export product categories with other countries are less than 1/5 of the total product categories in the industry. Fifth, we filter the country pairs whose Paasche price indexes are greater than the Laspeyres price indexes. Sixth, we omit the country pairs whose difference in the number of non-common export products between the two countries is greater than the quantity of common export products. The resulting data set can be used to estimate the industries’ IPIs of various countries relative to the benchmark country. The descriptions of the estimation characteristics are presented in Table 2. All the proportions of the estimated values lying between the Laspeyres and Paasche indexes are greater than 75% in Table 2, indicating that the estimated results of IPIs in the 14 industries are relatively reliable.

### 2.4. Measurement Results

Using the estimated IPIs above and taking the real effective exchange rates as the instrument for IPIs, the parameter estimates of Equation (9) by 2SLS are reported in Table 3. The results reveal that all the estimated coefficients of IPIs are significantly negative, in line with the theoretical expectations. Meanwhile, the partial F statistics for the first stage of 2SLS are all greater than 10, indicating that the instrument chosen in this study is valid. Based on the above estimation results, substituting the estimates of $\gamma_{s}$, $\zeta_{0s}^{ko}$ and $\zeta_{1s}^{ko}$ into Equation (10), we can calculate the export quality indexes of manufacturing industries from 1998 to 2015.

### 2.5. Model Specification

Based on the theoretical analysis, the following econometric model is constructed to empirically investigate the impact of environmental regulation on the export quality of China’s manufacturing industry:

(13) $$\ln Quality_{st}=\beta_{11}+\beta_{12}\ln ER_{st}+\beta_{13}Control_{st}+\delta_{s}+\delta_{t}+\varepsilon_{it},$$

where ln represents the natural logarithm applied to a variable to produce more stationary data; *Quality_st_* denotes the product quality of export in industry *s* in year *t*; *ER_st_* represents the intensity of environmental regulation in industry *s* at time *t*; and *Control* is a vector of industry characteristics including firm size in an industry (*Size*), capital–labor ratio (*Capital*), human capital (*HC*), and foreign investment (*FI*). $\delta_{s}$ denotes the industry fixed effect, which controls for unobserved heterogeneity, and $\delta_{t}$ denotes the year fixed effect, to control for the time trend. $\varepsilon_{st}$ is the random error term. If the results show that $\beta_{12}$ is significantly positive, environmental regulation mainly produces innovation offset effects, therefore promoting export quality upgrading. In contrast, if $\beta_{12}$ is significantly negative, environmental regulation principally exerts cost effects and inhibits export quality upgrading.

Furthermore, using a mediating effect model, we test the channels through which environmental regulation affects quality upgrading to more deeply explore the interrelation between environmental regulation and export quality. Based on the analysis of mechanism in Section 2.1, we choose process and product productivity as intermediary variables. Generally, the establishment of a mediating effect model involves three moves. First, make a regression analysis of the dependent variable with respect to the basic independent variable, i.e., Equation (13). Second, perform regression testing of the two intermediary variables with respect to the basic independent variable, respectively. Finally, set up a complex regression equation relating the dependent variable to the basic independent variables and the intermediate variables simultaneously. In short, the mediating effect model is comprised of Equation (13) and the following three equations:

(14) $$\ln\phi_{st}=\beta_{21}+\beta_{22}\ln ER_{st}+\beta_{23}Control_{st}+\delta_{s}+\delta_{t}+\varepsilon_{it},$$

(15) $$\ln\xi_{st}=\beta_{31}+\beta_{32}\ln ER_{st}+\beta_{33}Control_{st}+\delta_{s}+\delta_{t}+\varepsilon_{it},$$

(16) $$\ln Quality_{st}=\beta_{41}+\beta_{42}\ln ER_{st}+\beta_{43}\ln\phi_{st}+\beta_{44}\ln\xi_{st}+\beta_{45}Control_{st}+\delta_{s}+\delta_{t}+\varepsilon_{it},$$

where $\phi_{st}$ and $\xi_{st}$, respectively, denote the process and product productivity in industry *s* in year *t*. Equation (14) aims to examine the relationship between environmental regulation and process productivity, and Equation (15) is to test the link between environmental regulation and product productivity. If the results show that both $\beta_{22}$ and $\beta_{32}$ are significant, Equation (16) is estimated to examine whether environmental regulation and the two types of productivity are significantly related to export quality. If $\beta_{43}$ and $\beta_{44}$ are statistically significant as well, the mediating effects of process and product productivity on the relationship between environmental regulation and export quality are confirmed. In addition, if both $\beta_{12}$ and $\beta_{42}$ are statistically significant and $\beta_{42}$ is lesser than $\beta_{12}$, the two types of productivity play a partial mediating role. If $\beta_{42}$ is not significant, but both $\beta_{43}$ and $\beta_{44}$ are significant, there is a complete mediating effect.

### 2.6. Indicators of Econometric Regression

#### 2.6.1. Export Quality (Quality)

The quality indices of China’s sub-industries from 1998 to 2015, estimated in Section 2.2, Section 2.3 and Section 2.4, are used to measure the level of the export quality of China’s manufacturing industry.

#### 2.6.2. Environmental Regulation (ER)

For the measurement of environmental regulation, various methods are employed in the existing literature, but, at present, the most popular one is to adopt the comprehensive index of environmental regulation intensity. This involves taking the emission intensity of the different pollutants of an industry as the measure of the stringency of environmental regulation. Nevertheless, this method has two likely issues. First, due to the inconsistency of the main pollutants across industries, the indicators of different pollutant discharges need a weighted average adjustment. Second, considering the different attributes and characteristics among industries, different industries need to make different degrees of effort to meet the same environmental requirements for emissions. In order to solve the two problems above, we refer to the method of Wang et al. [41]. In addition, to construct the comprehensive index of the intensity of environmental regulation, allowing for the availability of data on pollutant discharges, this study selects the rate of compliance for industrial wastewater discharge standards, the removal rate of industrial SO_2_, the removal rate of industrial soot, the removal rate of industrial dust, and the ratio of industrial solid waste utilized.

First, each individual indicator of pollutant discharge is standardized as follows:

(17) $$PE_{sht}=\left(E_{sht}-Min\;E_{h}\right)/\left(Max\;E_{h}-Min\;E_{h}\right),$$

where $E_{sht}$ is the original value of the indicator for pollutant *h*; $\mathrm{Max}E_{h}$ and $\mathrm{Min}E_{h}$ represent the maximum and minimum values, respectively, of the indicator for pollutant *h* over the sample period; and $PE_{sht}$ is the standardized value of the indicator for pollutant *h*.

Second, we take a weighted average of the five indicators of pollutant discharge above. As the discharge of pollutants in different industries varies greatly, the weights are determined by:

(18) $$W_{sht}=\frac{E_{sht}}{\sum E_{sht}}/\frac{O_{st}}{\sum O_{st}},$$

where $O_{st}$ is the total industrial output, and $W_{sht}$ denotes the weight. Equation (18) indicates that the weight of pollutant *h* in industry *s* is determined by the relative emission intensity of pollutant *h.* Thus, it is beneficial for eliminating the impact of pollution emission intensity differences between industries.

Third, we use the operating expenses of pollution control facilities per unit of output as the measure of the pollution treatment costs ($fee_{st}$) of industries to deal with the discrepancy in effort expended between industries with different pollution intensity aiming to meet the same emission standard. The weight of pollution control costs in each industry is given by:

(19) $$wfee_{st}=fee_{st}/\sum fee_{st},$$

Finally, the intensity of environmental regulation can be measured by:

(20) $$ER_{st}=wfee_{st}\times \frac{1}{N}\sum W_{sht}\times PE_{sht},$$

where *N* represents the number of industries.

#### 2.6.3. Control Variables

In general, firms that produce high-quality products are more likely to have a large scale of production and use advanced equipment and skilled labor [1]. In addition, the export product quality of foreign-funded enterprises is higher than that of domestic enterprises [42]. Therefore, four control variables are added into our econometric model: firm size in an industry (*Size*), capital–labor ratio (*Capital*), human capital (*HC*), and foreign investment (*FI*). In this paper, firm size in an industry (*Size*) is defined as the average industrial output of each firm, measured by the ratio of total industrial output of an industry to the number of firms in this industry. Capital–labor ratio (*Capital*) is measured by the ratio of fixed asset investment in an industry to the number of industry employees. Human capital (*HC*) is evaluated by the proportion of scientific and technological personnel in each industry to the number of employees in that industry. Foreign investment (*FI*) is measured by the proportion of the gross industrial output of foreign-funded enterprises to the total gross industrial output of the industry.

#### 2.6.4. Mediating Variable

Process productivity (ϕ) mainly reflects the difference in variable cost between enterprises, so we use labor productivity as its proxy. Product productivity (ξ) shows the ability of enterprises to improve product quality with given fixed expenditures. However, due to a lack of information about the ability to produce quality, it is not easy to obtain a reliable direct estimate of product productivity. To address this problem, we use R&D efficiency to approximate the product productivity because R&D expenditure is an important part of enterprises’ fixed expenditures. The higher the R&D efficiency, the higher the product productivity of enterprises [43], as there is a strong positive correlation between the two.

### 2.7. Sources of Data

Data pertaining to total industrial output, fixed asset investment, number of enterprises, and number of employees in industries are derived from the China Industrial Economy Statistical Yearbook. The data on scientific and technological personnel in industries are retrieved from the China Science and Technology Statistics Yearbook. Relevant environmental data are obtained from the China Environmental Statistics Yearbook. Furthermore, considering that the industry classification in the China Environmental Statistics Yearbook has changed since 2002, we refer to the method of Liu and Xie [2] to integrate the data before and after 2002 into the same statistical specification. Additionally, these data are primarily based on CNIC 2002. In order to couple CNIC 2002 with ISIC Rev.4, we incorporate China’s 29 manufacturing sub-industries into the 14 manufacturing industries in Section 2.3 according to the correspondence in Table 1. Indicators and data sources for the main variables are listed in Table 4.

## 3. Results

### 3.1. Benchmark Regression Results

Before the regression estimation, we first examine whether the collinearity between variables is material. Although the panel data used in this study may control for individual differences to a certain extent, to reduce the collinearity, we still investigate the variance inflation factor (VIF) of each explanatory variable as a precautionary measure. All of them are less than 5, indicating that there is no serious collinearity in our econometric model. In addition, to control for possible heteroscedasticity in the model, we log-transform all variables to reduce the variance. Meanwhile, to further eliminate the influence of heteroscedasticity, *t*-statistics or *z*-statistics of all estimated parameters are obtained by clustering robust standard deviations, thereby ensuring the reliability of the results. To estimate the impact of environmental regulation on export quality, we adopt the panel fixed effect (FE) or random effect (RE) model for benchmark regression, and the Hausman test is used to determine the appropriate model. The benchmark regression results are reported in Table 5.

Model (1) In Table 5 is the benchmark model, which does not include any control variables and fixed effects, and thus separately examines the impact of environmental regulation on the export quality of China’s manufacturing industry. Model (1) shows that the estimated coefficient of environmental regulation (ln*ER*) is 0.237, which is positive at a significance level of 1%, indicating that environmental regulation can significantly promote the export quality upgrading of China’s manufacturing industry. For estimating the impact of environmental regulation more accurately, we introduce the industry fixed effect and the year fixed effect into Model (2). The results confirm that the estimated coefficient of environmental regulation remains significantly positive. Furthermore, control variables such as firm size in an industry (ln*Size*), capital–labor ratio (ln*Capital*), human capital (ln*HC*), and foreign investment (ln*FI*), are gradually added into Models (3)–(6). We find that the coefficients of environmental regulation are still positive at a significance level of 1% in these four models, and they display a slightly decreasing trend. These findings reveal that the positive effect of environmental regulation is quite stable.

In particular, in Model (6), the coefficient of environmental regulation is 0.203, indicating that every 1% increase in the intensity of environmental regulation is paralleled by a 0.203% rise in the export quality index of China’s manufacturing industry. Considering the reality of environmental protection in China, this result can be interpreted as follows: the Chinese government, in recent years, has revised certain laws and regulations regarding the environment, such as the Environmental Protection Law, the Air Pollution Prevention Law, the Energy Conservation Law, and the Water Law, and has put them into practice. With the increasing intensity of the environmental regulation and the gradual enhancement of public awareness of environmental protection, environmental regulation triggers R&D and innovation investment among manufacturing enterprises, thus promoting export product quality improvement.

In terms of control variables, in Models (3)–(6), the effect of firm size in the industry on the export quality is not significant, providing empirical evidence for the study of Hallak and Sivadasan [37], to some extent, who argue that firm size is not the primary cause of the quality difference among firms. In Models (4)–(6), the estimated coefficient of the capital–labor ratio is positive and significant at the 5% level; in Models (5)–(6), the estimated coefficient of human capital is also significantly positive. These results indicate that enterprises of higher-quality products need more intensive use of physical capital and highly skilled labor [44,45]. In Model (6), the estimated coefficient of foreign investment is significantly positive, indicating that foreign-funded enterprises play a significant role in the export quality upgrading of China’s manufacturing industry. Compared with local Chinese enterprises, the products of foreign-funded enterprises have lower domestic added value, higher proportion of processing trade, and higher quality of intermediate input, so they export higher-quality products. In addition, the technology spillover effect of foreign-funded enterprises is also conducive to the improvement of the product quality of local enterprises [46,47].

### 3.2. Mediation Test

Our investigation of the impact of environmental regulation on export quality finds that strict environmental regulation policies can significantly promote the export quality upgrading of China’s manufacturing industry. Therefore, we are interested to explore how this occurs. To answer this question, in this section, we adopt the mediating effect model constructed in Section 2.5 to empirically test whether process and product productivity mediate the relationship between environmental regulation and export quality.

Table 6 reports the estimated results of the mediating effect model. Model (1) in Table 6 is the estimation of the basic model (Equation (13)), and its results are completely consistent with Model (6) in Table 5. Model (2) and Model (3) in Table 6 are the estimations of Equations (14) and (15), respectively. In addition, the two mediating variables, process productivity (lnϕ) and product productivity (lnξ), are added to Equation (13), and the results are listed in Model (4) and Model (5). Furthermore, we add the two mediating variables into Equation (13) simultaneously, i.e., Equation (16), and the results are reported in Model (6).

Model (2) in Table 6 shows that the estimated coefficient of environmental regulation is −0.060, at a significance level of 1%, indicating that environmental regulation significantly inhibits the enhancement of process productivity in China’s manufacturing industry. This may be due to the fact that the compliance costs of environmental regulation add extra constraints on the production and operating activities of manufacturing enterprises, thus increasing the average costs per unit of output and reducing process productivity [25,27]. In contrast, in Model (3) the coefficient of environmental regulation is 0.195 and significant at the 1% level, suggesting that environmental regulation can significantly promote the improvement of product productivity in China’s manufacturing industry. This result is comparatively consistent with the theoretical analysis in Section 2; the innovation offset effect of environmental regulation encourages manufacturing enterprises to increase R&D input [19,21], thus improving both the ability to produce quality products and the product productivity [39,48].

As Models (4)–(6) show, both the estimated coefficients of lnϕ and lnξ are positive and significant, which means that industries with higher process or product productivity are more likely to export high-quality products. Meanwhile, it is also in line with the theoretical expectation. Compared with Model (1) in Table 6, the estimated coefficients and their *t*-statistics for environmental regulation in Model (4) and Model (5) both decrease when the two mediating variables are added into the basic model (Equation (13)), respectively. This finding provides preliminary evidence for the mediating effect of process and product productivity. Furthermore, when adding the two mediating variables into the basic model simultaneously, the estimated coefficient and its *t*-statistic for environmental regulation in Model (6) show a further decline. This result further proves that process and product productivity are two possible channels through which environmental regulation affects the export quality of China’s manufacturing industry.

To more rigorously confirm the mediating roles of process and product productivity in the relationship between environmental regulation and export quality, it is necessary to apply strict statistical tests. First, the following null hypotheses are constructed according to Equations (14)–(16): $H_{0}:\beta_{22}=0$;$H_{0}:\beta_{32}=0$;$H_{0}:\beta_{43}=0$;$H_{0}:\beta_{44}=0$. Second, these null hypotheses are statistically tested. If all of them are rejected, the mediating effect of process and product productivity is significant; otherwise, it is not significant. Models (2)–(6) show that $\beta_{22}$ in Equation (14), $\beta_{32}$ in Equation (15), and $\beta_{43}$ and $\beta_{44}$ in Equation (16) are all significant at least at the 5% level, indicating that process and product productivity play a significant mediating role in the link between environmental regulation and export quality for China’s manufacturing industry. However, the disadvantage of this statistical test is that the probability of making the second type of error is relatively high. In view of this, we reconstruct the null hypotheses and further test the mediating effect of process and product productivity: $H_{0}:\beta_{43}\beta_{22}=0$;$H_{0}:\beta_{44}\beta_{32}=0$. The mediating effect is proved to be significant only if both the null hypotheses are rejected; otherwise, it is not significant. Specifically, the standard deviation of $\beta_{43}\beta_{22}$ and $\beta_{44}\beta_{32}$ can be calculated using the method of Sobel [49]. Combined with the regression coefficients in Table 6, it is easy to obtain the statistics for the Sobel test: $Z_{\beta_{43}\beta_{22}}=-2.154$ and $Z_{\beta_{44}\beta_{32}}=2.373$, both significant at the 5% level. The results demonstrate convincingly that the mediating effects of process and product productivity do exist. In addition, both the coefficients of environmental regulation in Model (1) and Model (6) are significantly positive, and the latter (0.155) is less than the former (0.203), revealing that there is a partial mediating effect.

### 3.3. Analysis of Industry Heterogeneity

Some studies show that the influence of environmental policies on trade competitive advantage may have industry heterogeneity [2,29,30]. Due to the different attributes of manufacturing industries, specifically the difference in pollution emission intensity, industries vary in the efforts needed to meet the same environmental requirements. Therefore, referring to the method of Cheng et al. [50], we divide the manufacturing industries into two groups, pollution-intensive industries and clean industries, to test the heterogeneous impact of environmental regulation on the quality upgrading of China’s exports. Table 7 reports the results of the industry heterogeneity analysis.

Models (1)–(4) in Table 7 represent the estimates for pollution-intensive industries. Model (1) shows that the coefficient of environmental regulation is positive and significant, demonstrating that environmental regulation plays a significant role in promoting the export quality upgrading of pollution-intensive industries. The results also show that the coefficient of environmental regulation in Model (2) is negative and insignificant, while the coefficient in Model (3) is positive and significant. Meanwhile, the coefficients of lnϕ and lnξ in Model (4) are both significantly positive. It is, therefore, reasonable to infer that, for pollution-intensive industries, the channel through which environmental regulation promotes export quality upgrading is mainly product productivity.

Models (5)–(8) in Table 7 show the estimates for cleaner industries. In Model (5), the coefficient of environmental regulation is −0.087 and significant at the 5% level, revealing that environmental regulation has an inhibitory effect on the export quality upgrading of clean industries. Furthermore, as Models (6) and (7) indicate, the impact of environmental regulation on process productivity is significantly negative, while the impact on product productivity is positive, albeit insignificantly. Meanwhile, in Model (8), the coefficients of process productivity and product productivity are both positive and significant. These findings suggest that, for cleaner industries, the channel through which environmental regulation inhibits export quality upgrading is mainly process productivity.

### 3.4. Robustness Test

In the preceding analysis and discussion, we focus on the impact and its mechanism of environmental regulation on export quality, finding that environmental regulation significantly promotes the export quality upgrading of China’s manufacturing industry, and that process and product productivity mediate the relationship between environmental regulation and export quality. Although the industry fixed effect and the year fixed effect are controlled, the regression models above are still possibly subject to endogeneity caused by omitted variables. In addition, there are different measures of the key explanatory variable, environmental regulation, which may also affect the reliability of the results in this paper. Therefore, this paper conducts the robustness test from the following three aspects.

First, in order to address the potential endogeneity, as well as to take account of the dynamic characteristics of export quality, the SYS-GMM (System-Generalized method of moments) estimation is conducted. Panel A in Table 8 reports the results of the SYS-GMM estimation. The results of the AR (2) test show that none of the regression models can reject the null hypothesis that there is no second-order serial correlation of error terms. The results of the Sargan test also indicate that no regression model can reject the null hypothesis that the instrumental variables are valid. Therefore, the SYS-GMM estimation in this paper is valid. Furthermore, Panel A in Table 8 reveals that the impact of environmental regulation on export quality is still significantly positive, the mediating effect of product productivity (0.138 × 0.437) is distinctly greater than that of process productivity (−0.046 × 0.402), and the two mediating effects are in opposite directions. Hence, it is confirmed that there is no substantive change in the estimated results when considering endogeneity.

Second, we alter the measure of the key explanatory variable to re-examine the impact and its mechanism of environmental regulation on export quality. According to Lanoie et al. [26] and Cheng et al. [50], an industry’s expenditure on pollution control can objectively reflect the actual level of effort of emission reduction. Therefore, we take the proportion of the expenditure on industrial wastewater and waste gas treatment to total industrial output of an industry as an alternative measure of environmental regulation. As Panel B in Table 8 shows, the significance and magnitude of the coefficients of the key explanatory variable (environmental regulation) and the two mediating variables (process and product productivity) are substantially consistent with the prior estimated results, which further demonstrates the robustness of our empirical results.

Third, allowing for the lag effect of environmental regulation on export quality, as well as the endogenous problem due to the reverse causality between environmental regulation and export quality, the first-order lag of environmental regulation (ln*ER_t_*_-1_) is selected for regression. Panel C in Table 8 shows that there are no substantial changes in the coefficients of the key explanatory variable and the two mediating variables.

## 4. Discussion

To supplement existing theoretical and empirical studies, this study investigates the systematic relationship between environmental regulation and export competitiveness from the perspective of product quality. The findings suggest that environmental regulation has a significant and stable promoting effect on the export quality upgrading of China’s manufacturing industry. This is basically consistent with the research of Li and Chen [51] and Liu and Xie [2], who believe that the innovation offset effect of strict environmental regulation policies is conducive to improving the export competitive advantage of “Made in China”. It also demonstrates that China’s environmental regulation policies significantly support the strong Porter hypothesis [39,52,53].

Moreover, the results of mediation test not only fully verify the existence of the mediating effects, but also reflect that the directions of the mediating effects of process and product productivity are opposite to each other. Further calculation shows that the mediating effect of process productivity is −0.025 (−0.060 × 0.412), and the mediating effect of product productivity is 0.093 (0.195 × 0.477). Comparing them in absolute value terms, the mediating effect of product productivity is significantly greater than that of process productivity. This finding implies that the main effect of environmental regulation on China’s manufacturing industry is innovation offset, which is beneficial to export quality upgrading in general. It also lends support to the benchmark regression results above.

However, further analysis of industry heterogeneity reveals that the impact of environmental regulation on export quality and its mechanism show the significant heterogeneity of regulated industries. For pollution-intensive industries, environmental regulation plays a significant promoting role through the channel of product productivity, but, for clean industries, environmental regulation has an inhibitory effect through the channel of process productivity. A possible reason for this is that the government’s environmental policies of energy conservation and emission reduction put great pressure on enterprises in pollution-intensive industries, which in turn forces them to conduct technological innovation and quality upgrading in order to survive [2]. On the contrary, the intensity of environmental regulation implemented by the government on clean industries is so weak, and it fails to reach the threshold value of the Porter hypothesis. Consequently, environmental regulation mainly exerts a cost effect on clean industries [24], thereby reducing process productivity and inhibiting export quality upgrading.

Furthermore, our findings provide important enlightenment for the coordinated development of China’s ecological civilization and trade power. First, environmental regulation is an important driving force for the improvement of export quality. Therefore, the government should strengthen the intensity of environmental regulation within certain limits, forcing manufacturing enterprises to engage in clean technology innovation and improve product quality, thus promoting the transformation and upgrading of export trade. The government should raise the market access threshold for environmental standards, so that environmental regulation can play a screening role in enterprises entering the export market, thereby optimizing the reallocation of resources among enterprises.

Second, the government should design differentiated environmental regulation policies for industries with different pollution intensities. For pollution-intensive industries, the government can adopt environmental standards, emission limits, and other command-and-control environmental policy instruments to give full play to the role of environmental regulation in export quality upgrading, accelerate process improvement and product renewal, and realize the sustainable development of export trade. For clean industries, market-based environmental policy instruments, such as emission trading and environmental subsidies, can be used to stimulate enterprises to accelerate clean technology research and development, thereby meeting the environmental requirements of foreign markets and fostering new advantages in export competition.

In addition, the findings of this paper can also provide beneficial environmental policy implications to other developing countries. As a developing country, China inevitably faces the problem of how to deal with ecological deterioration and environmental pollution in the process of rapid industrialization [54]. We find that environmental regulation can not only promote cleaner production, pollution control and emission reduction, but also facilitate the high-quality development of the manufacturing industry. Therefore, developing countries, on the one hand, should formulate environmental policies appropriate to their own economic development level, thereby phasing out manufacturing enterprises with high pollution and low efficiency. On the other hand, developing countries should selectively attract investment and prudently treat the participation of foreign capital in pollution-intensive industries [55], so as to avoid becoming the pollution haven of developed countries.

However, there are some limitations in this study. We focus on the investigation of market competitiveness from the perspective of product quality, as well as its determinants, whereas the relationship between price and product quality is neglected. Our theoretical framework is constructed at the firm-product level, but we conduct empirical tests at the industry level due to the limited availability of micro-data. In addition, the impact of environmental regulation on export quality is a complex process, and there may be multiple mechanisms. In view of this, this paper should be considered as a preliminary analysis, and more detailed and in-depth research is still needed.

## 5. Conclusions

Most studies focus on the empirical investigation of the relationship between environment and trade, but they lack a systematic theoretical framework. To fill this gap, this paper constructs an analytical framework of export competitiveness from the perspective of product quality and reveals the theoretical mechanism of environmental regulation affecting export quality. Further, we empirically examine the impact of environmental regulation on the export quality of China’s manufacturing industry, as well as its possible mechanism. The findings are as follows. (1) Environmental regulation can significantly promote the export quality upgrading of the manufacturing industry. That is, China’s environmental regulation policies support the strong Porter hypothesis, to some extent, and every 1% increase in the intensity of environmental regulation is paralleled by a 0.203% rise in the quality index of China’s manufacturing industry. (2) Process and product productivity are two possible channels through which environmental regulation affects export quality, and their mediating effects are in opposite directions. In comparison, the mediating effect of product productivity is obviously greater than that of process productivity, indicating that environmental regulation mainly has an innovation offset effect on China’s manufacturing industry. (3) The impact of environmental regulation on export quality and its mechanism show the significant heterogeneity of regulated industries. For pollution-intensive industries, environmental regulation plays a significant promoting role through the channel of product productivity, but, for clean industries, environmental regulation has an inhibitory effect through the channel of process productivity.

## Figures and Tables

**Table 1:** Correspondence table between ISIC Rev.4 and China’s National Industrial Classification (CNIC) 2002.

Industry	Description	ISIC Rev.4	CNIC 2002
ind1	Food, beverage and tobacco	D10-12	13–16
ind2	Textile, clothing, leather and footwear manufacturing	D13-15	17–19
ind3	Wood and straw products	D16	20
ind4	Paper and printing products	D17-18	22–23
ind5	Energy products and chemicals	D19-21	25–28
ind6	Rubber and plastic products	D22	29–30
ind7	Non-metallic mineral products	D23	31
ind8	Base metal manufacturing	D24	32–33
ind9	Calendered metal manufacturing	D25	34
ind10	Other machinery and equipment manufacturing industry	D28	35–36
ind11	Computer, electronic and optical products	D26	40–41
ind12	Electrical equipment manufacturing	D27	39
ind13	Vehicle manufacturing	D29-30	37
ind14	Furniture and other manufacturing	D31-32	21, 24, 42

**Table 2:** The Estimation characteristics of IPIs.

Industry	Sample Country	Country Pair	Country Pair- Product	The Median Interval between the Paasche and Laspeyres Index	The Proportion of the Estimated Value Lyling between the Paasche and Laspeyres Index
ind1	22	212	6639	0.69	82.79%
ind2	22	210	9153	0.80	82.63%
ind3	18	133	1371	0.93	85.76%
ind4	19	148	2072	0.82	86.67%
ind5	21	180	6222	1.15	88.19%
ind6	19	154	2119	0.67	77.70%
ind7	19	153	2672	1.07	80.25%
ind8	16	108	2145	1.00	82.14%
ind9	20	182	4690	1.16	90.71%
ind10	22	221	7391	0.93	88.05%
ind11	22	210	5399	0.96	87.20%
ind12	18	146	2120	1.00	87.84%
ind13	19	136	1688	0.91	83.57%
ind14	22	177	2648	0.65	84.17%

Note: To ensure that the benchmark country is consistent in all industries, Canada is selected as the benchmark country. The interval between the Paasche and Laspeyres index is computed by the logarithm of the Laspeyres index minus that of the Paasche index. The proportion of the estimated value lying between the Paasche and Laspeyres index is mainly used as a fitting goodness indicator.

**Table 3:** The result of the two-stage least squares (2SLS) estimate.

	**ind1**	**ind2**	**ind3**	**ind4**	**ind5**	**ind6**	**ind7**
$\;\ln\;{\hat{P}}_{st}^{ko}$	−0.119 ***	−0.073 ***	−0.014 ***	−0.016 ***	−0.136 ***	−0.038 ***	−0.025 ***
	(−3.252)	(−2.940)	(−2.988)	(−3.624)	(−3.006)	(−3.865)	(−2.970)
R^2^	0.834	0.832	0.935	0.732	0.848	0.914	0.866
Partial F	61.13	18.72	12.38	104.56	35.62	22.57	37.23
observations	396	396	324	342	378	342	342
	**ind8**	**ind9**	**ind10**	**ind11**	**ind12**	**ind13**	**ind14**
$\;\ln\;{\hat{P}}_{st}^{ko}$	−0.099 ***	−0.045 ***	−0.108 ***	−0.054 **	−0.049 **	−0.167 **	−0.036 ***
	(−3.365)	(−2.901)	(−2.834)	(−2.362)	(−2.295)	(−2.330)	(−3.007)
R^2^	0.827	0.740	0.764	0.875	0.782	0.814	0.848
Partial F	25.90	19.91	11.39	16.95	15.12	22.07	13.99
observations	288	360	396	396	324	342	396

Notes: t-statistics in parentheses. ** and *** represent 5% and 1% significant levels, respectively. Due to space limitations, estimates of country fixed effects and time trends are not reported.

**Table 4:** Indicators and data sources for the main variables.

Variable Category	Variable	Indicator	Data Source
Dependent variable	*Quality*	Export quality index	Measurement in Section 2.2, Section 2.3 and Section 2.4
Core independent variable	*ER*	Comprehensive index of intensity of environmental regulation	China environmental statistics yearbook
Control variables	*Size*	The ratio of total industrial output to the number of firms in the industry	China Science and Technology Statistics Yearbook China Industrial Economy Statistical Yearbook
	*Capital*	The ratio of fixed asset investment to the number of employees in the industry	
	*H* *C*	The proportion of scientific and technological personnel to the number of employees in the industry	
	*FI*	The proportion of the gross industrial output of foreign-funded enterprises to the total gross industrial output of the industry	
Mediating variables	*φ*	Labor productivity	
	*ξ*	R&D efficiency	

**Table 5:** Benchmark regression results.

Variable	Model (1)	Model (2)	Model (3)	Model (4)	Model (5)	Model (6)
ln*ER*	0.237 ***	0.229 ***	0.218 ***	0.210 ***	0.206 ***	0.203 ***
	(4.228)	(4.142)	(3.925)	(3.917)	(3.906)	(3.852)
ln*Size*			0.048	0.052	0.056	0.046
			(1.303)	(1.083)	(1.117)	(1.031)
ln*Capital*				0.094 **	0.086 **	0.079 **
				(2.103)	(2.284)	(2.148)
ln*Human*					0.033 ***	0.025 **
					(3.236)	(2.167)
ln*FDI*						0.063 ***
						(3.007)
Constant	3.285 *	2.547 **	1.832 **	4.295 ***	2.572 **	2.593 **
	(1.774)	(2.063)	(2.184)	(3.832)	(2.375)	(2.055)
Industry fixed effect	N	Y	Y	Y	Y	Y
Year fixed effect	N	Y	Y	Y	Y	Y
R-squared	0.376	0.383	0.388	0.394	0.396	0.399
F/Wald	83.74 ***	117.32 ***	142.03 ***	76.76 ***	315.29 ***	173.40 ***
Hausman	3.41	2.59	58.35 ***	53.61 ***	48.03 ***	52.47 ***
Estimation method	RE	RE	FE	FE	FE	FE
Observations	252	252	252	252	252	252

Notes: Robust *t*-statistics or *z*-statistics are in parentheses; *, **, and *** indicate significance at 10%, 5%, and 1% levels, respectively; Y means the industry or year fixed effect is controlled, whereas N represents that is not controlled.

**Table 6:** Results of mediation test.

Variable	ln*Quality*	lnϕ	lnξ	ln*Quality*	ln*Quality*	ln*Quality*
	Model (1)	Model (2)	Model (3)	Model (4)	Model (5)	Model (6)
ln*ER*	0.203 ***	−0.060 ***	0.195 ***	0.187 ***	0.183 ***	0.155 ***
	(3.852)	(−4.291)	(3.562)	(3.720)	(3.739)	(3.382)
ln*Size*	0.046	0.033 *	0.081	0.029	0.053	0.036
	(1.031)	(1.904)	(1.340)	(1.052)	(1.118)	(1.007)
ln*Capital*	0.079 **	0.057 ***	0.017 **	0.066 ***	0.070 **	0.059 **
	(2.148)	(3.604)	(2.183)	(3.427)	(2.223)	(2.105)
ln*Human*	0.025 **	0.092 **	0.014 ***	0.023 **	0.017 **	0.011 **
	(2.167)	(2.362)	(3.773)	(2.153)	(2.044)	(2.060)
ln*FDI*	0.063 ***	0.082 *	0.024 **	0.060 **	0.058 ***	0.056 ***
	(3.007)	(1.837)	(2.303)	(1.312)	(3.320)	(3.221)
lnϕ				0.456 ***		0.412 **
				(3.301)		(2.483)
lnξ					0.515 ***	0.477 **
					(3.927)	(2.235)
Constant	2.593 **	−0.927 ***	10.599 **	−2.629 ***	1.285 **	7.437 ***
	(2.055)	(−5.781)	(2.089)	(−6.134)	(2.327)	(4.520)
Industry fixed effect	Y	Y	Y	Y	Y	Y
Year fixed effect	Y	Y	Y	Y	Y	Y
R-squared	0.399	0.270	0.314	0.413	0.407	0.418
F/Wald	173.40 ***	108.35 ***	81.97 ***	111.64 ***	77.03 ***	93.68 ***
Hausman	52.47 ***	42.09 ***	46.63 ***	69.14 ***	56.33 ***	47.02 ***
Estimation method	FE	FE	FE	FE	FE	FE
Observations	252	252	252	252	252	252

Notes: Robust *t*-statistics or *z*-statistics are in parentheses; *, **, and *** indicate significance at 10%, 5%, and 1% levels, respectively; Y means that the industry or year fixed effect is controlled.

**Table 7:** Results of industry heterogeneity analysis.

Variable	ln*Quality*	lnϕ	lnξ	ln*Quality*	ln*Quality*	lnϕ	lnξ	ln*Quality*
	Model (1)	Model (2)	Model (3)	Model (4)	Model (5)	Model (6)	Model (7)	Model (8)
ln*ER*	0.461 ***	−0.034	0.325 ***	0.373 ***	−0.087 **	−0.068 ***	0.104	−0.070 *
	(3.724)	(−1.190)	(3.036)	(3.142)	(−2.142)	(−3.392)	(0.969)	(−1.823)
ln*Size*	0.041	0.038	0.077	0.032	0.048	0.030	0.087	0.039
	(0.827)	(1.294)	(1.014)	(1.271)	(1.113)	(1.175)	(1.255)	(1.108)
ln*Capital*	0.071 **	0.053 **	0.022 **	0.056 **	0.083 **	0.051 ***	0.016 **	0.067 **
	(2.207)	(2.330)	(2.169)	(2.085)	(2.150)	(3.074)	(2.118)	(2.100)
ln*Human*	0.021 *	0.095 **	0.017 ***	0.017 **	0.027 **	0.090 **	0.013 **	0.015 **
	(1.895)	(2.183)	(3.289)	(2.104)	(2.307)	(2.208)	(2.160)	(2.085)
ln*FDI*	0.055 **	0.080 *	0.022 *	0.043 **	0.074 ***	0.085 **	0.029 **	0.062 ***
	(2.113)	(1.860)	(1.804)	(2.028)	(3.576)	(2.330)	(2.137)	(3.285)
lnϕ				0.364 *				0.475 **
				(1.843)				(2.377)
lnξ				0.482 **				0.470 **
				(2.125)				(2.254)
Constant	6.654 ***	2.943 ***	−3.599 ***	0.590 ***	2.081 ***	−1.860 ***	0.254 ***	1.047 ***
	(4.650)	(3.832)	(−5.269)	(4.105)	(4.412)	(−3.942)	(5.038)	(4.149)
Industry fixed effect	Y	Y	Y	Y	Y	Y	Y	Y
Year fixed effect	Y	Y	Y	Y	Y	Y	Y	Y
R-squared	0.387	0.277	0.316	0.404	0.381	0.280	0.342	0.415
F/Wald	202.76 ***	82.20 ***	194.65 ***	142.04 ***	232.81 ***	94.87 ***	105.62 ***	143.07 ***
Hausman	37.20 ***	28.43 ***	31.52 ***	25.64 ***	50.02 ***	3.49	2.83	34.30 ***
Estimation method	FE	FE	FE	FE	FE	RE	RE	FE
Observations	144	144	144	144	108	108	108	108
Sample category	pollution-intensive industries				clean industries			

Notes: Robust *t*-statistics or *z*-statistics are in parentheses; *, **, and *** indicate significance at 10%, 5%, and 1% levels, respectively; Y means that the industry or year fixed effect is controlled.

**Table 8:** Results of the robustness test.

Variable	ln*Quality*	lnϕ	lnξ	ln*Quality*
	Model (1)	Model (2)	Model (3)	Model (4)
Panel A: SYS-GMM estimation				
ln*ER*	0.127 ***	−0.046 **	0.138 ***	0.104 **
	(3.167)	(−2.308)	(3.042)	(2.273)
lnϕ				0.402 **
				(2.245)
lnξ				0.437 **
				(2.066)
Constant	11.475 ***	9.434	31.159	2.124 **
	(4.478)	(1.247)	(0.873)	(2.552)
Control Variables	Y	Y	Y	Y
Industry fixed effect	Y	Y	Y	Y
Year fixed effect	Y	Y	Y	Y
AR (1)	[0.008]	[0.036]	[0.017]	[0.022]
AR (2)	[0.266]	[0.174]	[0.409]	[0.320]
Sargan test	[0.921]	[0.648]	[0.511]	[0.836]
Panel B: Altering the measure of key variable				
ln*ER*	0.313 **	−0.082 **	0.175 ***	0.284 **
	(2.295)	(−2.150)	(3.127)	(2.076)
lnϕ				0.348 **
				(2.316)
lnξ				0.409 **
				(2.157)
Constant	−4.186 *	6.056 ***	2.613 ***	0.182 ***
	(−1.805)	(5.069)	(3.861)	(4.642)
Control Variables	Y	Y	Y	Y
Industry fixed effect	Y	Y	Y	Y
Year fixed effect	Y	Y	Y	Y
R-squared	0.368	0.305	0.329	0.404
F/Wald	73.15 ***	168.34 ***	156.05 ***	102.26 ***
Hausman	56.21 ***	39.98 ***	43.50 ***	45.17 ***
Estimation method	FE	FE	FE	FE
Observations	252	252	252	252
Panel C: Using the first-order lag of environmental regulation for regression				
ln*ER_t-_*_1_	0.255 ***	−0.043 **	0.162 ***	0.197 ***
	(3.684)	(−2.241)	(3.308)	(3.172)
lnϕ				0.430 ***
				(3.194)
lnξ				0.461 ***
				(4.285)
Constant	0.938 ***	5.611 **	−2.041 ***	7.547 ***
	(6.001)	(2.165)	(−4.760)	(5.483)
Control Variables	Y	Y	Y	Y
Industry fixed effect	Y	Y	Y	Y
Year fixed effect	Y	Y	Y	Y
R-squared	0.355	0.263	0.307	0.394
F/Wald	159.44 ***	101.03 ***	130.57 ***	97.62 ***
Hausman	41.39 ***	54.07 ***	49.81 ***	63.20 ***
Estimation method	FE	FE	FE	FE
Observations	238	238	238	238

Notes: Robust *t*-statistics or *z*-statistics are in parentheses; the *p* values of the respective statistics are in square brackets; *, **, and *** indicate significance at 10%, 5%, and 1% levels, respectively; Y represents that the industry or year fixed effect is controlled, or that all the control variables are included.

## Appendix A. The Derivation of the Profit Function

Using Equation (2), the revenue of firm *i* is given by:

(A1) $$r_{ij}=p_{ij}q_{ij}=\frac{\lambda_{ij}^{\sigma -1}}{p_{ij}^{\sigma -1}}\frac{E}{P},$$

According to Hallak and Sivadasan [37], the operative profits that are the difference between firm revenue and variable costs equal r/σ. Therefore, the operative profits ($\pi^{o}$) of firm *i* can be expressed as:

(A2) $$\pi_{ij}^{o}=\frac{r_{ij}}{\sigma }=\frac{1}{\sigma }{\left(\frac{\lambda_{ij}}{p_{ij}}\right)}^{\sigma -1}\frac{E}{P},$$

Then, subtracting fixed costs of production and fixed trade costs from the operative profits, we obtain the post-entry profits of firm *i*:

(A3) $$\pi_{ij}=\pi_{ij}^{o}-F_{ij}-f_{x}=\frac{1}{\sigma }{\left(\frac{\lambda_{ij}}{p_{ij}}\right)}^{\sigma -1}\frac{E}{P}-F_{ij}-f_{x},$$

The above is the profit function (Equation (5)).
